# Investigating efficacy of two brief mind–body intervention programs for managing sleep disturbance in cancer survivors: a pilot randomized controlled trial

**DOI:** 10.1007/s11764-012-0252-8

**Published:** 2013-01-22

**Authors:** Yoshio Nakamura, David L. Lipschitz, Renee Kuhn, Anita Y. Kinney, Gary W. Donaldson

**Affiliations:** 1 Utah Center for Exploring Mind–Body Interactions (UCEMBI), Pain Research Center, Department of Anesthesiology, School of Medicine, University of Utah, 615 Arapeen Drive, Suite 200, Salt Lake City, UT 84108 USA; 2 Pain Research Center, Department of Anesthesiology, School of Medicine, University of Utah, Salt Lake City, UT USA; 3 Cancer Control and Population Sciences Program, the Huntsman Cancer Institute, and Department of Internal Medicine, School of Medicine, University of Utah, Salt Lake City, UT USA

**Keywords:** Mind–body bridging, Mindfulness meditation, Cancer survivors, Sleep disturbance, Depression, Quality of life

## Abstract

**Purpose:**

After completing treatment, cancer survivors may suffer from a multitude of physical and mental health impairments, resulting in compromised quality of life. This exploratory study investigated whether two mind–body interventions, i.e., Mind–Body Bridging (MBB) and Mindfulness Meditation (MM), could improve posttreatment cancer survivors’ self-reported sleep disturbance and comorbid symptoms, as compared to sleep hygiene education (SHE) as an active control.

**Methods:**

This randomized controlled trial examined 57 cancer survivors with clinically significant self-reported sleep disturbance, randomly assigned to receive MBB, MM, or SHE. All interventions were conducted in three sessions, once per week. Patient-reported outcomes were assessed via the Medical Outcomes Study Sleep Scale and other indicators of psychosocial functioning relevant to quality of life, stress, depression, mindfulness, self-compassion, and well-being.

**Results:**

Mixed effects model analysis revealed that mean sleep disturbance symptoms in the MBB (*p* = .0029) and MM (*p* = .0499) groups were lower than in the SHE group, indicating that both mind–body interventions improved sleep. In addition, compared with the SHE group, the MBB group showed reductions in self-reported depression symptoms (*p* = .040) and improvements in overall levels of mindfulness (*p* = .018), self-compassion (*p* = .028), and well-being (*p* = .019) at postintervention.

**Conclusions:**

This study provides preliminary evidence that brief sleep-focused MBB and MM are promising interventions for sleep disturbance in cancer survivors. Integrating MBB or MM into posttreatment supportive plans should enhance care of cancer survivors with sleep disturbance. Because MBB produced additional secondary benefits, MBB may serve as a promising multipurpose intervention for posttreatment cancer survivors suffering from sleep disturbance and other comorbid symptoms.

**Implications for Cancer Survivors:**

Two brief sleep-focused mind–body interventions investigated in the study were effective in reducing sleep disturbance and one of them further improved other psychosocial aspects of the cancer survivors’ life. Management of sleep problems in survivors is a high priority issue that demands more attention in cancer survivorship.

## Introduction

The experience of cancer and its treatment can take a heavy toll on cancer patients, who, following treatment, may continue to suffer from many physical and psychological concerns. Fatigue, depression, cognitive impairment, and sleep disturbance are found to be related to compromised quality of life (QOL), decreased treatment adherence, and increased cancer-related morbidity [1]. In particular, sleep disturbance has been recognized as a persisting problem that is not always addressed effectively in posttreatment care [2–4]. Cancer survivors need better options for alleviating sleep disturbance and improving health-related QOL.

Sleep disturbance often includes reduced sleep efficiency, prolonged latency to falling asleep, and increased wake time during the night. Recent data indicate that a high percentage (19–30 %) of cancer patients report sleep problems [5, 6]. Almost 20 % of posttreatment breast cancer patients reported more than 6 months of chronic insomnia [1], continuing for several years after diagnosis in a large number of breast cancer patients [7]. Furthermore, insomnia is a powerful predictor of cancer-related fatigue [1]. Standard pharmacological treatment options for sleep problems include medications such as benzodiazepines and benzodiazepine receptor agonists [8]. While these may provide some symptom relief, tolerance may develop, and long-term use can cause fragmented sleep patterns and overreliance on medication to facilitate sleep onset [8]. Furthermore, medication side effects may be troublesome.

Basic and clinical science evidence supports the premise that mind–body interventions can enhance patients’ healing and recovery, and increase well-being and autonomy [9]. What we term mindfulness-based interventions (MBIs) are mind–body interventions that focus on the power of mental training in regulating health conditions [10]. MBIs utilize mental training to facilitate awareness, attention, intention, and attitude [11]. In our view, MBIs comprise interventions such as Mindfulness-Based Stress Reduction (MBSR) [12, 13], Mindfulness-Based Cognitive Therapy (MBCT) [14, 15], and a recent addition, Mind–Body Bridging (MBB) [16]. These MBIs may help treat and manage conditions such as cancer, chronic pain, sleep disturbance, and depression (see Shapiro and Carlson [17] for an overview).

Several recent studies investigated the efficacy and utility of MBSR in managing persisting symptoms in cancer patients and posttreatment survivors [7, 18–25]. The findings from these studies are positive and promising, suggesting that MBSR is effective for managing persisting symptoms in breast and prostate cancer patients. More recently, a number of systematic reviews and meta-analyses [26–29] of MBSR and MBI for cancer patients and survivors have appeared, further documenting that MBSR in particular and MBI in general are effective in controlling symptoms and improving QOL in cancer patients and survivors.

Nonetheless, MBSR’s efficacy in specifically improving sleep remains to be demonstrated conclusively. In a systematic review of clinical studies of MBSR targeting sleep [30], seven studies were identified. The review reported that the lack of standardized outcome measures precludes pooling of results for quantitative data analysis and that sleep report measures varied (standardized scales, single item, and sleep diaries). Four studies (all uncontrolled) found that MBSR significantly improved measures of sleep quality or duration, while three studies found no statistically significant difference between treatment and control conditions.

Another recent randomized controlled trial study directly compared MBSR against a standard pharmacotherapy treatment for chronic primary insomnia [31]. The authors reported that MBSR improved sleep onset latency measured by actigraphy (8.9 min) as well as large significant improvements measured by various self-report sleep measures. Britton and her colleagues [32] examined whether mindfulness meditation (MM) was associated with changes in objectively measured polysomnographic (PSG) sleep profiles and whether changes in PSG sleep were related to subjectively reported changes in sleep and depression. They found that according to PSG sleep data, mindfulness meditation practice was associated with several indices of increased cortical arousal, including more awakenings and stage 1 sleep and less slow-wave sleep relative to wait-list controls, in proportion to amount of meditation practice. This study underscores potential complex relationships between meditation practice and objective/subjective sleep measures.

We recently evaluated MBB, in a prospective randomized controlled trial with veterans who suffered from clinically significant self-reported sleep disturbance [33]. The results from our study supported the efficacy of brief sleep-focused MBB in improving not only sleep disturbance but also self-reported comorbid posttraumatic stress disorder (PTSD) symptoms in veterans. Clinicians working at the VA Salt Lake City Health Care System have used MBB in their clinical practice dealing with a variety of mental health conditions and reported anecdotally that MBB seemed effective for managing conditions ranging from PTSD, depression, substance abuse to sleep disturbance, as well as for improving health-related quality of life in patients with wide ranging conditions.

In light of this preliminary evidence reported above, we reasoned that MBB might be useful in managing sleep and other comorbid symptoms in cancer survivors. We also wanted to examine the efficacy of MBB together with another MBI such as Mindfulness Meditation (MM), which is perhaps one of the most critical components of MBSR and other MBIs (see Chiesa and Serretti [34] for reviews of mindfulness meditation and its benefits). As an active control condition, we included Sleep Hygiene Education (SHE).

Thus, our exploratory study with cancer survivors employed a three-armed randomized controlled parallel group study design. MBB and MM have both similarities and differences. Both MM and MBB highlight awareness practices as core skills in the programs. However, MM obviously includes meditation practice, while MBB does not. MBB is focused on developing experiential bridging skills and utilizing Mind–Body Mapping (an exercise designed to make explicit people’s often implicit “requirements” of situations; see [35] for more details), while MM does not explicitly include such an exercise for identifying requirements. Given these shared features and key differences, comparison of the two interventions may offer useful pilot data for clarifying the nature of potential therapeutic benefits from these two programs. On the basis of clinicians’ anecdotal reports supporting potential usefulness of MBB for wide-ranging conditions, we also wanted to investigate the potential impacts of these sleep-focused MBIs on common comorbid symptoms and psychosocial indicators. Thus, our study included several secondary outcome measures, as a means of more fully understanding the new intervention programs’ potential impacts [36].

We hypothesized that MBB and MM would improve self-reported sleep problems and reduce other behavioral or psychological symptoms persisting into the postcancer treatment period. More specifically, we hypothesized that MBB and MM would be more effective than SHE in managing sleep problems in cancer survivors. The study targeted cancer survivors without requiring specific types of cancer for at least two reasons. First, health-related QOL compromised by sleep disturbance and other behavioral or psychological symptoms may be more or less comparable across subjects with different cancer types [37]. Second, some level of symptom burden in cancer survivorship is highly prevalent across different types of cancer [38]. Thus, this study served as an exploratory pilot study for establishing the broad efficacy of MBB and MM for supportive care of cancer survivors, by exploring potential impacts of these interventions on sleep (the primary endpoint) and several secondary outcome measures (e.g., depression, well-being, and self-compassion).

## Methods

### Participants and recruitment

We recruited participants from various cancer clinics (Huntsman Cancer Institute, University of Utah), a cancer support center (Cancer Wellness House), and cancer support events (Survivors at the Summit), and through flyers and newspaper advertisements. Participants were male and female, 18–75 years old. We conducted the study from July 2009 through April 2010 at Cancer Wellness House, a nonprofit organization offering support for those living with cancer, surviving cancer, and their family members. The Institutional Review Board at the University of Utah approved the study protocol.

#### Inclusion criteria

Since this was an exploratory study, we included survivors of any type of cancer. Cancer survivor was defined as any patient who had completed active cancer treatment. To qualify, participants must have completed active cancer treatment (surgery, chemotherapy, or radiation therapy) *at least 3 months prior to attending the first study session*. However, they could be on adjuvant hormone therapy (e.g., Tamoxifen, Lupron, or aromatase inhibitors), sleep medication, or other medications (exceptions listed in exclusion criteria, below). Participants exhibited *self-reported sleep disturbance*, assessed by a validated sleep questionnaire, the Medical Outcomes Study Sleep Scale (MOS-SS) [39]; to be eligible to participate in the study, participants needed to score at least 35 on the MOS-SS Sleep Problems Index II subscale (SPI-II). Scores of 35 and above suggest a high probability of a clinically significant sleep disturbance. No specialized or medical diagnosis of insomnia was undertaken or required. Any type of sleep disturbance or problem was included. Participants spoke and understood English.

#### Exclusion criteria

The following criteria resulted in exclusion from the study: (1) any underlying severe or untreated psychopathology or psychiatric illness (e.g., schizophrenia); (2) cognitive impairment; (3) neurologic disorder; (4) dementia; (5) taking antipsychotic medication, immunosuppressants and/or corticosteroids; (6) having a metastatic disease; and (7) previous exposure to MBB or MM or MBSR or MBCT.

### Study design

We conducted a prospective, randomized study investigating the effects of the three short-term interventions on sleep in cancer survivors. All interventions consisted of three 2-h sessions, provided once a week for three consecutive weeks. Using computer-generated numbers, we randomly assigned participants to either one of the two interventions selected for each treatment cycle out of the three intervention programs investigated in the study: the SHE program (active control), MBB, and MM. The number of consented participants available at a specific time point determined the size of the groups in each cycle. Since the interventions were held at the same location, we assigned each participant to one of the interventions when they arrived for their first class. We randomized participants in randomly generated blocks of two interventions per cycle (except for the last cycle), maintaining balance of assignments over the seven cycles.

### Interventions

#### Sleep hygiene education program

The control intervention was SHE, in which participants received educational classes informing them how to change their habits to improve sleep, and what to do if they had concerns about sleep quality. The program was based on the Huntsman Online Patient Education (HOPE) Guide, “Insomnia: patient symptom information” (http://www.hopeguide.org/hope/). Topics included (but were not limited to): “Why would I have insomnia?” (caused by some medications, stress, anxiety, or depression, pain), “When should I call my doctor?” (worries or concerns about sleeping or have questions about insomnia), “What can I do to get a better night’s sleep?” (diet, exercise, supportive care, getting ready to sleep), “What should I avoid to get a better night’s sleep?” (tips on behaviors that can negatively influence sleep quality), and “What should I know about medicine for sleep problems?” (side-effects and mechanisms of sleep medications and other medications affecting sleep).

After the SHE instructor explained each HOPE recommendation, participants had an opportunity to ask questions and discuss their sleep issues. Participants were encouraged to follow the SHE recommendations on a daily basis. SHE classes were presented by a licensed clinical social worker with extensive expertise in cancer supportive care.

#### Mindfulness Meditation program

MM was based on MBSR [40], which is a longer program teaching awareness and mindfulness skills, including basic meditation practice and yoga. MBSR was developed and implemented to treat persistent and elevated levels of stress, sleep disturbance, and other behavioral problems in both healthy subjects and those with clinical illnesses (for a meta-analysis review, see Grossman et al. [41]). Mindfulness may be defined as paying attention in a particular way, on purpose, in the present moment, and nonjudgmentally [40]. The goal of MBSR is to provide participants with experiential tools and mindfulness practices that help them become more aware of how the mind (including thoughts, emotions and physical sensations) works and relates to external environments. MBSR programs focusing on sleep symptoms previously have used a 6-week long format [7, 21], twice as long as our MM program. It should be noted here that the MM program was not intended to cover all the elements included in the standard MBSR program.

To create a 3-week MM program for our study, we specifically and selectively focused on various forms of mindfulness meditation (e.g., sitting, walking, and body scan). Our MM course included the following: during mindfulness meditation practices at weekly group meetings, fundamental mindfulness meditation skills (breath awareness, awareness of thoughts and emotions), body scans, walking meditation, and forgiveness meditation were reviewed and taught. Group members initially were asked to introduce themselves and include, if they wished, something about their cancer experience and sleep difficulties. Thereafter, they were asked to talk about their cancer only as related to what issues came up during the practices or processes presented. Attention was given to how this differentiates the MM program from a traditional cancer support group. Time was devoted in sessions 2 and 3 to group discussions concerning the implementation of moment-to-moment awareness in the participants’ lives, allowing for the opportunity for them to learn from one another about how to integrate mindfulness throughout the day. Discussion of issues concerning sleep was focused on the participants’ experience related to sleep disturbance, having utilized the processes associated with mindfulness throughout the day.

Home practice assignments were at each individual’s discretion, given that participants were not told in advance that there would be extensive homework assignments (as would be the case in MBSR programs). Participants were free to utilize the MBSR meditation CDs covering body scan and breath meditation, as their schedules allowed, noting that some practice would facilitate a more successful outcome. (The MM program did not involve any focus on yoga practice, and participants were instructed to work with their health care provider/physical therapist prior to any utilization of the MBSR yoga CDs.) Participants were also assigned a writing exercise wherein they were asked to write about a concern that was causing them to feel stressed, with a focus on including emotional content and expressing themselves as fully as possible. Handouts for the course included the CDs for mindfulness meditation, a copy of the book, *Full catastrophe living*, a printout on “The practice of mindfulness” taken from the book, and stress reduction tips. MM was taught by a licensed clinical social worker/oncology social worker who is an experienced MBSR instructor and has years of meditation experience.

#### Mind–Body Bridging program

A summary of MBB appears in a few other publications discussing this novel program [33, 35]. Briefly, MBB is a mind–body intervention that may be beneficial for a wide range of mental and physical health problems. MBB teaches awareness skills that help individuals recognize and become aware of a dysfunctional mind–body state characterized by heightened self-centeredness, as indicated by ruminative thoughts, involuntary constriction of awareness, body tension, and impaired mental or physical functioning. MBB also teaches mind–body “mapping” exercises, which are designed to reveal thought patterns known as “requirements” and to link them to bodily states experienced at the time when “requirements” are identified. Requirements are expectations about how people and the world should be at particular moments (an example of a requirement is “I should cope with my cancer more effectively” or “I should not have any symptoms”). Identification of “requirements” is the key component of the MBB program. However, unlike cognitive behavioral therapy-based programs, MBB does not strive to identify maladaptive thought patterns and does not attempt to change them into more adaptive ones. When requirements are not fulfilled, people may develop ruminative storylines that in turn may lead to dysfunctional mind–body states. Using awareness practices and defusing requirements, over time and with practice, MBB practitioners learn to expand their awareness, deal more effectively with daily life’s challenges, and foster more balanced, harmonious mind–body states. In this way, MBB carries awareness practices one critical step further by addressing the underlying cause of the resistances to clarity, i.e., mental afflictions caused by an individual’s fixed idea of who she/he is, known as the “Identity System” or “I-System” in MBB teaching language. When requirements are not satisfied, the I-System gets activated and produces a self-centered mind–body state. When this happens, it can impede a person’s natural functioning. Increased awareness of the I-System may contribute to MBB’s therapeutic usefulness. MBB techniques are easy to learn and benefits may accrue rapidly (see Tollefson et al. [35] for more detailed descriptions of a general MBB program). In our previous study [33], as compared with SHE, MBB significantly reduced sleep disturbance in veterans after two weekly sessions. For the present study, the third session was added to the program used in the previous study in order to be responsive to common issues characteristic of cancer survivors.

The study MBB training covered: identifying what, from MBB’s perspective, may be a critical component in persistent sleep problems; learning to use MBB tools, such as exercises to reduce daytime stress; increasing self-awareness; addressing sleep issues pertinent to cancer survivors; and practicing MBB on a daily basis. The MBB training program was taught by a licensed clinical social worker who is a certified MBB instructor.

#### Adherence to programs

Participants in all groups were asked to apply the techniques and the information they learned during the study and the 2-month period leading to the follow-up assessment. In the MM group, they were encouraged to listen to CDs and read portions of the two books used in the course. In the MBB group, they were asked to complete mind–body map exercises from the textbook used. In the SHE group, they were asked to review periodically what they learned from the SHE program.

### Study procedures

Potential participants contacting the study team completed a screening questionnaire, which covered sleep patterns, demographics, cancer history (type of cancer and treatment), medical history, and medications/supplements used. If a person passed this screening phase, the study team contacted that person’s health care provider (with the participant’s signed consent) to verify that their mental health and cancer treatment status did not preclude participation in the study. Participants agreed to participate in the study by signing an informed consent form.

Participants were assessed for study outcome measures using self-report questionnaires, at prerandomization (baseline), at postintervention, and at 2-month follow-up. They also completed exit surveys at postintervention and follow-up. We invited subjects who did not complete the study to participate in the follow-up assessment.

### Outcome measures

#### Primary measures

##### Medical Outcomes Study Sleep Scale [39]

The MOS-SS is a validated 12-item scale providing an index of sleep problems *over the past week* and incorporating six subscales: (1) sleep disturbance, (2) sleep adequacy, (3) daytime somnolence, (4) snoring, (5) waking up short of breath with a headache, and (6) quantity of sleep. Two additional subscales evaluate composite sleep problems based on the above subscales: Sleep Problems Indexes I and II (SPI-I and SPI-II). SPI-II was the main inclusion and outcome measure used in this study, as it is a composite score reflecting sleep disturbance, sleep adequacy and somnolence.

The MOS-SS is similar to the Pittsburgh Sleep Quality Index (PSQI) developed around the same time, but PSQI is not validated for a 1-week time frame and thus was judged to be suboptimal for our study.

We assessed sleep, using MOS-SS at *six time points,* including: *two baselines* (1) at least 1 week before the interventions started and (2) immediately prior to the beginning of the first intervention session and *four assessments at post randomization* (3) at week 2 (before the second intervention session), (4) at week 3 (before the third intervention session), (5) postintervention, and (6) follow-up.

MOS-SS has been employed to investigate disrupted sleep in a variety of cancer populations [42, 43]. In the present study, Cronbach’s alpha coefficient for SPI-II was .72, which is comparable to that reported by Hays et al. [39].

The following primary and secondary measures described below were assessed at *three time points:* (1) pre (baseline), (2) post, and (3) follow-up.

##### Functional Assessment of Cancer Therapy—General [44]

Since we are interested in how the mind–body interventions may help cancer survivors, we assessed the programs’ relative efficacy using Functional Assessment of Cancer Therapy—General (FACT-G), a nonspecific cancer-based measure of QOL/well-being. FACT-G includes 27 items and encompasses 4 different indices of well-being: physical, social/family, emotional, and functional well-being [44], and it has been used to evaluate cancer-related QOL in many studies [45–49]. In the present study, Cronbach’s alpha coefficient for FACT-G total score was .92.

##### Perceived Stress Scale [50, 51]

The Perceived Stress Scale (PSS) measures the degree to which participants consider situations to be stressful, based on the extent to which they perceive their lives to be unpredictable, uncontrollable, or overloaded. We used the 10-item version, which has maximum reliability. The PSS has been previously used to evaluate impacts of an MBSR program in cancer survivors [52]. In the present study, Cronbach’s alpha coefficient for PSS total score was .92.

#### Secondary measures

##### Center for Epidemiological Studies-Depression Scale [53]

The Center for Epidemiological Studies-Depression Scale (CES-D) comprises 20 items and is one of the most common screening tests for the presence of depressive symptoms. CES-D has been used in many different studies of adult cancer survivors [48, 49, 54, 55]. In the present study, Cronbach’s alpha coefficient for CES-D total score was .92.

##### Impact of event scale [56, 57]

The Impact of event scale (IES) is a standardized self-report 15-item questionnaire that examines intrusive and avoidant thoughts and actions that a person experiences related to cancer. The IES has been used to assess impacts of an MBSR program in cancer survivors [52] and in other studies to investigate quality of life in long-term adult cancer survivors [45, 48]. In the current study, Cronbach’s alpha coefficient for IES total score was .91.

##### Five-Facet Mindfulness Questionnaire [58]

The Five-Facet Mindfulness Questionnaire (FF-MQ) includes 39 items and assesses five distinct, interpretable facets of mindfulness, including (1) observing, (2) describing, (3) acting with awareness, (4) nonjudging of inner experience, and (5) nonreactivity to inner experience. We computed a total mindfulness score, which we used to analyze the degree to which the interventions cultivated mindfulness in the participating cancer survivors. The FF-MQ was used in a study investigating the effects of an MBSR program in posttreatment cancer patients [52]. In the current study, Cronbach’s alpha coefficient for FF-MQ total score was .92.

##### Self-Compassion Scale [59]

This scale comprises 26 items and is used to measure self-compassion, an emotionally positive attitude that can protect against the negative consequences of self-judgment, isolation, and rumination (such as in depression). Self-compassion entails three main components: (1) self-kindness (being kind and understanding toward oneself in instances of pain or failure, rather than being harshly self-critical); (2) common humanity (perceiving one’s experiences as part of the larger human experience, rather than seeing them as separating and isolating); and (3) mindfulness (holding painful thoughts and feelings in balanced awareness, rather than over-identifying with them). The Self-Compassion Scale (SCS) was recently used in an MBSR study [60], and a subscale of the SCS was used in breast cancer survivors to understand the role of self-kindness [61]. In the current study, Cronbach’s alpha coefficient for the total score was .96.

##### WHO Well-Being Index [62]

Study participants filled out the WBI, which was developed and validated by the World Health Organization Collaborating Centre in Mental Health. This five-item index covers positive and negative aspects of emotional functioning [63]. In the current study, Cronbach’s alpha coefficient for the total score was .88.

##### Positive and Negative Affect Schedule [64]

Emotional experience can be reliably divided into the two categories of positive affect (PA) and negative affect (NA), which are consistent across many different dimensions (populations, languages, cultures, time frames, and response formats) [65]. A validated self-report measurement with 20 items combines these two factors in the Positive and Negative Affect Schedule (PANAS), comprising two 10-item scales for PA and NA [64]. The PANAS has been employed in several studies of long-term adult cancer survivors [45, 46, 54, 55, 66]. In the current study, Cronbach’s alpha coefficient for negative affect was .91 and that for positive affect was .89.

#### Other measures

We collected other information that may have affected outcomes, including age, gender, site of cancer and whether metastatic or nonmetastatic, date of cancer diagnosis and duration of cancer condition, and any additional interventions, such as adjuvant cancer treatment, supplements, and herbal treatments used by participants (see Table 1). We collected these as part of the screening process prior to the start of the study, but since this was an exploratory study, we did not stratify participants based on any of these variables.

**Table 1:** Demographics, medical history, and baseline self-report data for participants by treatment

	SHE	MBB	MM
Demographics			
Age (year)	51.6 (10.7)^ab^	55.4 (9.6)	50.8 (9.10)
Female	14	13	16
Male	4	6	4
Medical history			
Breast cancer	9	8	14
Other cancers^c^	10^d^	13^d^	6
Metastatic cancer	3	3	3
Median years since cancer diagnosis	4 years, 2 months	3 years 8 months	2 years 10 months
Reporting previous diagnosis of insomnia	6	8	8
Reporting previous diagnosis of clinical depression	6	6	7
Medications at baseline			
Sleep medications	5	4	5
Antidepressants	9	7	9
Any prescription medications	18	15	19
Any supplements/herbal remedies	16	17	17
Baseline measures^e^			
Sleep problems index II (SPI-II) score (MOS-SS)	54.94 (18.31)	58.01 (14.64)	63.33 (12.70)
Total sleep time (TST, h)	6.33 (1.18)	6.13 (1.08)	6.45 (1.19)
Depression rating (CES-D)	20.83 (12.65)	20.05 (11.56)	21.30 (10.30)

^a^SD in parentheses; all variables reflect number of subjects, unless indicated

^b^At baseline for age, the three groups did not differ significantly from one another (*p* = .308)

^c^Type of cancer, including ovarian, endometrial, testicular, prostate, lung, melanoma, ependymona, leukemia, kidney, lymphomas (non-Hodgkin’s, CNS), skin carcinoma, brain, thyroid, and peritoneal

^d^More than one cancer site in some individuals

^e^At prerandomization baseline, the three groups did not significantly differ from one another (SPI, *p* = .244; TST, *p* = .685; CES-D, *p* = .943)

### Statistical analyses

The data analyses evaluated: (1) whether the mind–body programs provided greater benefit for sleep than did the active control condition and (2) whether the mind–body programs provided greater additional benefit for other self-reported measures of coexisting symptoms or psychosocial indicators than did the active control intervention.

#### MOS-SS (primary outcome and endpoint)

To evaluate these objectives, we used mixed effects linear models under maximum likelihood to estimate the level and change of treatment benefit in the three groups, adjusted for baseline differences. The primary endpoint of the study based on the MOS-SS SPI II was the overall benefit from baseline, i.e., *cumulative changes over the four postrandomization periods* comprising week 2 (assessed before the beginning of the second session), week 3 (assessed before the beginning of the third session), post, and follow-up. We controlled for baseline differences using the prerandomization measures (baseline) as covariates. In line with the usual analysis of covariance (ANCOVA) strategy that is strongly recommended for analyzing clinical trial outcomes by Frison and Pocock [67], we emphasize that conditioning on baseline covariates performs statistical matching on the prerandomization scores and ensures that postrandomization treatment arm comparisons are free of baseline differences. A significant *F* test for the treatment arms indicates that it is unlikely that the adjusted population means are exactly equal. While the overall *F* tests are informative, since there are three groups in our exploratory study, they do not provide insight into the magnitudes of treatment effects. Given this, we constructed customized contrasts among the treatment vs. control arms (i.e., MBB vs. SHE and MM vs. SHE) using the overall postrandomization averages (comprising week 2, week 3, post, and follow-up), adjusted for baseline, to estimate the cumulative benefit of the three intervention programs individually and relative to one another.

#### Other outcomes measures

All other primary and secondary self-report questionnaires were assessed at three time points: pre, post, and follow-up. In all these analyses, we employed a mixed effects model ANCOVA with prerandomization (baseline) score as a covariate and with treatment arm and postintervention assessment time as categorical factors. As noted above, in line with the usual ANCOVA strategy, conditioning on baseline covariates performs statistical matching on the prerandomization scores and ensures that postrandomization treatment arm comparisons are free of baseline differences. For each self-report outcome measure, the analysis estimated overall treatment arm benefit conditional on assessment time. Just as in the primary outcome measure, to gain insight into the magnitudes of treatment effects, we further constructed customized contrasts among the treatment vs. control arms (i.e., MBB vs. SHE and MM vs. SHE) using the overall postintervention averages (post and follow-up) adjusted for baseline, to estimate the cumulative benefit of the three intervention programs individually and relative to one another.

The mixed effects analysis model generalizes conventional baseline-adjusted ANCOVA by more flexibly and accurately incorporating the statistical dependence arising with repeated observation of the same individuals. We used an objective criterion, the minimal value of the Bayesian Information Criterion, to determine the most parsimonious fit to alternative covariance structures of the repeated observations, retaining in all cases the full treatment arm-by-period factorial for the fixed effects. Mixed effects analyses invoke an “intent-to-treat” treatment of missing data by including every observation of the dependent measure. No observations are discarded, and no data are imputed. Rather, the algorithm chooses parameter estimates that generate the highest probability for all the data observed under the model assumptions.

## Results

### Attendance

**Fig. 1:**
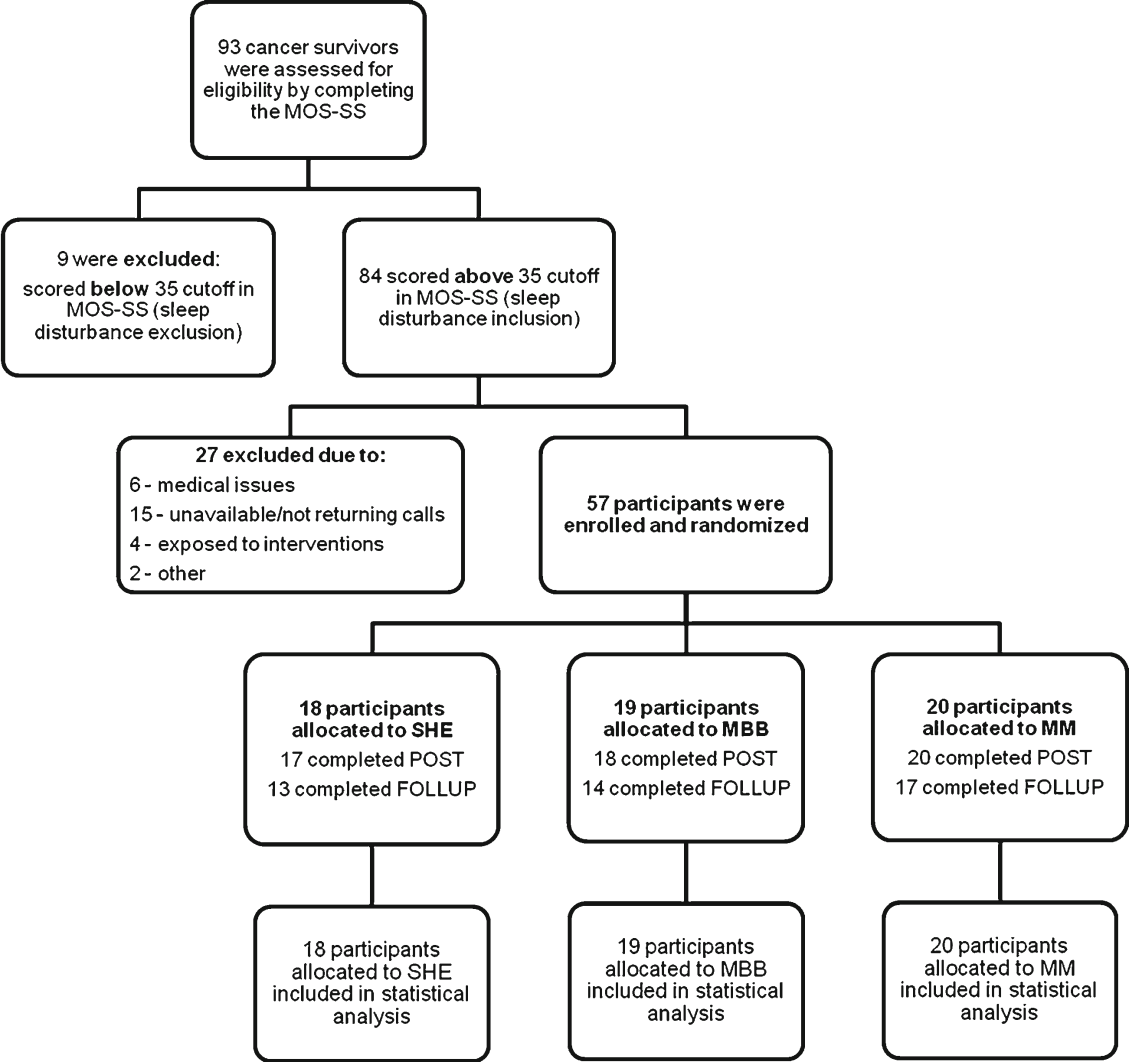
Study flow, described in accordance with the CONSORT Guidelines

Following the CONSORT recommendations, Fig. 1 depicts the number of individuals screened, qualified, and enrolled in the study, as well as retention numbers at post and follow-up, for each intervention. Of the 57 participants who enrolled (18 in SHE, 19 in MBB, 20 in MM), a total of 55 completed the interventions and postintervention questionnaires [17 in SHE (94.4 %), 18 in MBB (94.7 %), and 20 in MM (100 %)], and 44 completed the 2-month follow-up [13 in SHE (72.2 %), 14 in MBB (73.7 %), and 17 in MM (85 %)]. Only two participants missed one of the three classes. All except two participants completed postassessment; however, these two completed the follow-up.

### Demographics and baseline characteristics

Table 1 shows demographics and baseline measures, including cancer diagnosis and medications used by the 57 study participants before randomization. Participants were predominantly non-Hispanic white female and male; two female and one male participants were Hispanic. Female was the predominant gender, and female breast cancer survivors comprised the greatest number of participants (Table 1). Baseline characteristics were reasonably well-balanced among the three groups. Differences in baseline dependent measures were adjusted directly in the analysis.

### Outcome measures

#### Primary outcomes

##### MOS-SS Sleep Problems Index 2

**Fig. 2:**
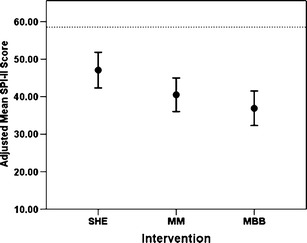
Primary outcome indicator, the adjusted means of MOS-SS Sleep Problems Index II (SPI-II), averaged over all time points in the three groups, with 95 % confidence interval (CI). Here, “adjusted” means “adjusted for baseline scores,” allowing the outcome indicators to be directly compared with one another. Both MM and MBB were found to be lower than SHE (*p* = .0499 and *p* = .0029 respectively). Note that the CIs depicted here are not those CIs actually used in the linear mixed model analysis. A *dashed horizontal line* in represents the mean baseline covariate value, representing a common baseline reference across the three groups

Cumulative mean scores, comprising the four postrandomization data points and adjusted for baseline differences, are displayed in Fig. 2. A dashed horizontal line in Fig. 2 represents the mean baseline covariate value representing a common reference point across the three treatment groups. In the ANCOVA, comparison of the error covariance structures supported the compound symmetry (or random intercept) model. The overall *F* test for arm was significant (*p* = .011), indicating that the average levels differed across treatment arms at postrandomization. The indicator of effect size in the linear mixed model ($R_{\beta}^2$) expressing the effect size of fixed effects in the linear mixed model [68] was estimated to be .13, reflecting the magnitude of treatment effects across the three groups. The estimated mean scores comprising the four postrandomization data points, adjusted for baseline, and 95 % confidence intervals (CIs) for each of the three treatment groups are presented under “Overall” in Table 2. The customized contrasts were MBB vs. SHE, *p* = .0029; MM vs. SHE, *p* = .0499, respectively. Table 3 presents effect sizes for pair-wise comparisons (MBB vs. SHE and MM vs. SHE), and $R_{\beta}^2$ corresponding to the magnitude of differences among the treatment groups, which are estimated to be analogous to Cohen’s d. In addition, all treatment interventions showed benefit in relation to the baseline covariate (59.19), with mean improvements (i.e., decreases from baseline) in self-reported sleep scores of 22.29 for MBB, 18.70 for MM, and 12.11 for SHE (all *p* < 0.001).

**Table 2:** Adjusted means (and 95 % CIs) of MOS-SS SPI-II scores and other secondary measures for comparisons among SHE, MBB, and MM

Scale	SHE	MBB	MM
MOS-sleep scale SPI-II			
Overall^a^	47.08 (42.33–51.88)	36.89 (32.30–41.48)	40.49 (36.02–44.96)
Week2	49.96 (44.55–55.37)	44.78 (39.56–50.01)	46.65 (41.52–51.79)
Week3	44.82 (38.93–50.71)	39.89 (34.09–45.68)	41.81 (36.22–47.40)
Post	50.04 (43.27–56.80)	32.94 (26.37–39.50)	41.29 (34.97–47.61)
Follow-up	43.52 (35.53–51.52)	29.97 (22.27–37.68)	32.20 (25.05–39.35)
CES-D total score	16.97 (13.59–20.35)	12.03 (8.75–15.31)	16.32 (13.20–19.44)
FF-MQ total score	129.30 (123.31–135.30)	139.51 (133.68–145.33)	133.27 (127.68–138.87)
Subscales			
Acting with awareness	25.55 (23.75–27.34)	27.82 (26.07–29.56)	24.67 (22.99–26.36)
Observing	27.86 (26.47–29.26)	29.92 (28.57–31.27)	29.47 (28.18–30.77)
SCS total score	82.09 (77.62–86.56)	89.15 (84.81–93.48)	86.44 (82.28–90.59)
WBI total score	14.16 (12.74–15.58)	16.54 (15.16–17.92)	15.24 (13.92–16.57)

^a^Overall = average of four scores at four different time points, which was defined as the primary outcome measure for the primary analysis

**Table 3:** Cohen’s effect sizes for pair-wise comparisons and $R_{\beta}^2$ for 3-group comparison for primary and secondary outcome measures

	Cohen’s effect size (*d*) estimates		Overall effect size
	MBB vs. SHE	MM vs. SHE	$R_{\beta}^2$
MOS-SS SPI-II	1.06	0.70	.13
CES-D	.44	.06	.09
FF-MQ	.52	.21	.10
SCS	.32	.20	.09
WBI	.52	.28	.10

**Fig. 3:**
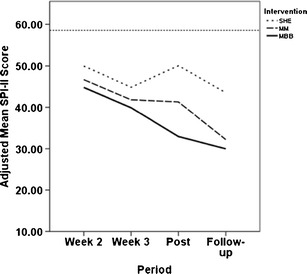
Estimated mean scores of SPI-II adjusted for baseline scores from pre, for SHE (*dotted line*), MBB (*solid line*), and MM (*dashed line*), as a function of assessment times (week 2, week 3, post, and follow-up). Here, “adjusted” means “adjusted for baseline scores,” allowing them to be directly compared with one another. Therapeutic benefits in MM and MBB continued to improve over time, while that in SHE leveled off after the completion of the study sessions. A *dashed horizontal line* in represents the mean baseline covariate value, representing a common baseline reference across the three groups

The adjusted means and 95 % CIs of the three groups at weeks 2 and 3 are presented in Table 2. Further inspection of these values indicated that during the active treatment period, there was no differential benefit immediately across the three groups. The adjusted means and 95 % CIs of the three groups at post and at follow-up are presented in Table 2. The customized contrasts at post were: MBB vs. SHE, *p* = .001 and MM vs. SHE, *p* = .065, respectively. The customized contrasts at follow-up were MBB vs. SHE, *p* = .018, and MM vs. SHE, *p* = .040, respectively. Figure 3 presents all adjusted means from the three groups over time.

For the other MOS-SS subscales, in accordance with greater improvements as indexed by SPI-II in sleep in the MBB and MM treatment groups, some subscales (adequacy, disturbance, and somnolence) revealed differences across the three interventions (see Table 4, statistics not shown). Table 4 shows unadjusted means and standard deviations of SPI-II and other subscales from the three groups at baseline, post and follow-up. Table 5 presents a pooled within-group covariance matrix with variances in diagonal and correlations off-diagonal for SPI-II. Table 6 presents unadjusted means and standard deviations of SPI-II from the three groups at six time points. All these data were used in the statistical analyses reported above. Data from Tables 5 and 6 should facilitate a systematic review and meta-analysis of MBIs in the future.

**Table 4:** Unadjusted means (and SDs) of MOS-SS subscale scores by treatment

		SHE	MBB	MM
MOS-SS subscale	Period			
Sleep disturbance	Baseline^a^	59.44 (25.90)	64.47 (21.50)	69.25 (17.90)
	Post	46.84 (23.64)	32.57 (20.32)	46.69 (23.81)
	Follow-up	39.13 (16.85)	31.79 (24.09)	37.50 (24.59)
Snoring	Baseline	35.29 (32.04)	34.12 (26.23)	34.00 (38.44)
	Post	30.00 (37.24)	28.24 (29.21)	33.68 (42.19)
	Follow-up	33.85 (38.63)	28.57 (31.10)	28.24 (33.95)
Awaken with shortness of breath/headache	Baseline	28.89 (29.28)	17.89 (20.97)	30.00 (34.64)
	Post	16.47 (29.36)	12.22 (24.87)	13.00 (20.80)
	Follow-up	23.08 (30.38)	5.71 (12.22)	7.06 (14.04)
Somnolence	Baseline	41.11 (24.44)	42.46 (21.68)	42.33 (13.90)
	Post	37.25 (23.69)	25.56 (19.57)	26.00 (17.69)
	Follow-up	30.77 (28.74)	24.29 (18.78)	26.27 (12.58)
Sleep adequacy	Baseline	35.56 (21.75)	25.26 (19.82)	21.00 (17.14)
	Post	34.71 (24.52)	52.78 (21.64)	36.00 (21.86)
	Follow-up	43.85 (27.25)	51.43 (22.14)	48.24 (24.04)
Sleep problems index I	Baseline	52.41 (18.11)	56.84 (13.03)	62.33 (13.12)
	Post	45.29 (17.84)	32.04 (14.51)	43.17 (15.76)
	Follow-up	40.77 (18.72)	31.19 (15.45)	35.10 (18.60)
Sleep problems index II	Baseline	54.94 (18.31)	58.01 (14.64)	63.33 (12.70)
	Post	47.22 (16.21)	32.13 (14.81)	43.64 (17.44)
	Follow-up	40.81 (18.64)	31.59 (16.95)	36.01 (17.52)
	Baseline	6.33 (1.18)	6.13 (1.08)	6.45 (1.19)
Sleep duration (hr.)	Post	6.41 (1.57)	6.97 (1.09)	7.00 (1.21)
	Follow-up	6.92 (1.55)	6.79 (1.05)	7.09 (1.75)

^a^For MOS-SS Sub-scales, baseline score reflects the value based on the first prerandomization assessment

**Table 5:** MOS-SS Sleep Problems Index-II (SPI-II) subscale indicating a pooled within-group covariance matrix with variances in diagonal and correlations off-diagonal

	Pre 1	Pre 2	Week 2	Week 3	Post	Follow-up
Pre1	(221.50)	.85	.64	.55	.39	.44
Pre2		(194.78)	.64	.51	.34	.39
Week2			(207.22)	.62	.44	.45
Week3				(219.33)	.72	.55
Post					(245.88)	.65
Follow-up						(285.45)

**Table 6:** Unadjusted means (and SDs) of MOS-SS SPI-II by treatment at different time points

	SHE	MBB	MM
Pre1	54.94 (18.31)	58.01 (14.64)	63.33 (12.70)
Pre2	54.57 (17.04)	57.08 (14.94)	61.42 (10.63)
Week2	47.61 (14.32)	44.15 (15.34)	49.00 (14.67)
Week3	42.47 (12.54)	39.23 (18.00)	44.16 (14.88)
Post	47.22 (16.21)	32.13 (14.81)	43.64 (17.44)
Follow-up	40.81 (18.64)	31.59 (16.95)	36.01 (17.52)

The proportions of cancer survivors whose MOS-SS scores fell and remained below the cutoff point for significant sleep disturbance (MOS-SS SPI-II score of 35 as used in the eligibility assessment) after completion of the intervention programs were as follows: At post, the proportions were 29.4 % below the cutoff for SHE, 66.7 % below the cutoff for MBB, and 45.0 % below the cutoff for MM. At follow-up, the proportions were 46.2 % below the cutoff for SHE, 57.1 % below the cutoff for MBB, and 52.9 % below the cutoff for MM.

In summary, all treatments provided apparent benefit from preintervention baselines. Figure 3 graphically illustrates that SHE was effective while the intervention sessions were ongoing, but MM and MBB were effective longer after the completion of the study program. It appears that MBB may have maintained a constant relative superiority of 3–4 points over MM throughout the duration of the study.

##### Functional Assessment of Cancer Therapy—General

Total FACT-G scores increased by 3–7 points in the three interventions (Table 7). Both active interventions showed benefit, with mean improvements of 7.47 (*p* = .002) for MBB and 6.0 (*p* = .010) for MM, while the SHE control group produced an improvement of 3.94 (*p* = .116), from the baseline covariate value (72.40). However, ANCOVA revealed no significant difference across the groups (*p* = .625). Comparisons of mean scores for each FACT-G subscale indicated that there is no difference among the groups (data not shown). Nonetheless, the observed overall improvements in the three groups are comparable to the minimally important difference, the magnitude of 3–7 points change reported for the FACT-G overall score [69]. This suggests that all interventions positively impacted QOL as measured by FACT-G.

**Table 7:** Unadjusted means (and SDs) of self-report outcome measures by treatment

		SHE	MBB	MM
Outcome measure	Period			
FACT-G	Baseline	71.59 (20.76)	73.12 (15.52)	70.92 (15.16)
	Post	76.50 (15.88)	78.54 (17.65)	74.88 (16.14)
	Follow-up	79.54 (14.81)	81.20 (18.10)	78.17 (16.45)
PSS total score	Baseline	17.56 (7.49)	18.53 (6.10)	20.20 (7.35)
	Post	15.41 (7.83)	14.82 (4.29)	16.90 (4.85)
	Follow-up	15.08 (6.33)	11.85 (5.03)	15.29 (5.63)
CES-D total score	Baseline	20.83 (12.65)	20.05 (11.56)	21.30 (10.30)
	Post	16.65 (9.85)	12.67 (8.85)	18.25 (10.86)
	Follow-up	17.15 (12.78)	10.57 (10.11)	15.18 (10.17)
IES total score	Baseline	33.20 (16.18)	37.06 (14.57)	33.89 (16.15)
	Post	27.12 (15.78)	32.11 (16.95)	34.16 (15.23)
	Follow-up	32.23 (17.59)	25.59 (14.33)	31.75 (13.45)
FF-MQ total score	Baseline	125.06 (22.32)	128.32 (15.65)	124.45 (21.69)
	Post	125.71 (24.44)	139.61 (12.66)	129.50 (20.58)
	Follow-up	133.54 (24.64)	141.00 (11.99)	135.18 (20.43)
SCS total score	Baseline	86.28 (26.48)	80.11 (20.14)	80.30 (21.89)
	Post	82.35 (23.66)	84.33 (17.59)	82.70 (19.33)
	Follow-up	88.77 (25.35)	89.86 (22.37)	87.88 (19.82)
WBI total score (well-being)	Baseline	11.11 (4.99)	11.53 (4.54)	11.25 (4.54)
	Post	13.41 (4.77)	16.39 (4.16)	14.35 (3.65)
	Follow-up	15.92 (4.25)	17.00 (3.14)	15.76 (4.34)
PANAS-negative affect	Baseline	21.22 (9.96)	20.53 (6.78)	20.85 (7.39)
	Post	17.47 (8.52)	16.78 (6.28)	17.35 (6.12)
	Follow-up	19.23 (9.45)	15.57 (6.85)	16.88 (6.21)
PANAS -positive affect	Baseline	28.44 (7.76)	27.84 (6.70)	29.00 (8.02)
	Post	29.24 (7.80)	31.11 (7.36)	30.75 (6.55)
	Follow-up	31.77 (6.47)	34.14 (7.20)	33.76 (6.30)

##### Perceived Stress Scale

The adjusted means of PSS 10 decreased in MBB (4.77), MM (3.01), and SHE (2.71) from the baseline covariate value of 18.60. Table 7 presents unadjusted means of PSS across the three groups. The mixed effects ANCOVA indicated that the three groups did not differ from one another (*p* = .279). The magnitude of change observed in the MBB group seems comparable to that (4.7 from pre to post) reported in one recent study of MBSR for cancer survivors [70] or those (5.7 from pre to post or 3.8 improvement relative to the wait-list control) reported in another recent study of MBSR for cancer patients [52].

#### Secondary outcomes

##### Center for Epidemiological Studies-Depression rating scale—total score

**Fig. 4:**
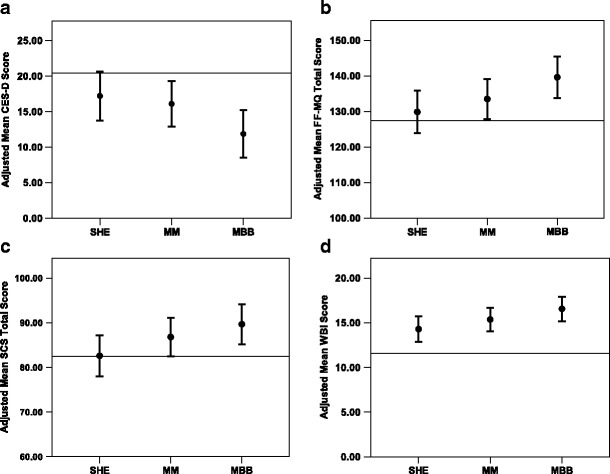
Effects of the three sleep interventions on: **a**) depression symptoms (CES-D), **b**) mindfulness (FF-MQ), **c**) self-compassion (SCS), and **d**) well-being (WBI). Estimated means, adjusted for baseline scores from Pre, for each scale are presented for SHE, MBB, and MM, with 95 % Confidence Interval (CI). Here, “adjusted” means “adjusted for baseline scores,” allowing them to be directly compared with one another. Note that the CIs depicted in Figure 4**a**, 4**b**, 4**c**, and 4**d** are not those CIs actually used in linear mixed model analysis. In all scales, MBB was different from SHE at post-intervention, indicating that MBB led to a reduced level of self-reported depression symptoms (4**a**) and enhanced levels of mindfulness, self-compassion, and well-being (4**b**, 4**c**, 4**d**). A dashed horizontal line in each Figure represents the mean baseline covariate value of each scale, representing a common baseline reference across the three groups

All treatment interventions showed apparent benefit from baseline levels, with mean improvements of 8.58 (*p* = .001) for MBB, 4.36 (*p* = .008) for MM, and 3.25 (*p* = .064) for SHE, in overall postintervention level. The magnitudes of these improvements are larger or comparable to what was reported (4.7 improvement from pre to post) in a recent feasibility study of an MBSR program for early stage breast cancer survivors [70]. Mixed effects ANCOVA of CES-D yielded *p* = .078 and $R_{\beta}^2$ = .088, overall. The adjusted means and 95 % CIs of the three groups are presented in Table 2. The customized contrasts were MBB vs. SHE, *p* = .040, and MM vs. SHE, *p* = .776, respectively. Figure 4a shows these adjusted means graphically (see Table 7 for unadjusted means and standard deviations of CES-D scores).

This suggests that MBB was more effective than SHE in reducing self-reported depression symptoms (see Table 3 for estimated effect sizes involving the CES-D). It is important to recognize that all three study interventions were focused on specifically improving sleep. Nonetheless, the data suggest that MBB reduced self-reported depression symptoms even though the MBB program did not explicitly address depression.

##### Impact of event scale

IES measures, as indicated by adjusted mean scores, decreased in MBB (6.43), MM (2.13), and SHE (2.9), respectively. Table 7 presents unadjusted means of IES across the three groups. The ANCOVA indicated that the groups did not differ from one another (*p* = .543). These observed reductions are comparable to those reported in another recent study of MBSR for cancer patients (approximately 2–3 point reductions in MBSR group and approximately 1 point reduction in the wait-list control)[52].

##### Five-facet Mindfulness Questionnaire

As shown in Fig. 4b, mindfulness in the MM and MBB groups seemed to increase at postrandomization. ANCOVA on total FF-MQ scores yielded *p* = .056 and $R_{\beta}^2$ = .101, overall. The adjusted mean total mindfulness scores (comprising all mindfulness subscales) and 95 % CIs of the three groups are presented in Table 2. The customized contrasts were MBB vs. SHE, *p* = .018, and MM vs. SHE, *p* = .335, respectively. Figure 4b presents these adjusted means graphically. Table 7 presents unadjusted means and standard deviations of FF-MQ. This suggests that MBB was more effective than SHE in increasing self-reported mindfulness (see Table 3 for estimated effect sizes involving the FF-MQ).

Since it is advantageous to assess separately the effects of the interventions on subscales of FF-MQ, we examined each subscale independently. For the FF-MQ subscale “acting with awareness,” the ANCOVA yielded *p* = .036, overall. The adjusted mean scores and 95 % CIs of the three groups are presented in Table 2. The customized contrasts were MBB vs. SHE, *p* = .075, and MM vs. SHE, *p* = .480, respectively. For the FF-MQ subscale “observing,” the ANCOVA yielded *p* = .093, overall. The adjusted mean scores and 95 % CIs of the three groups are presented in Table 2. The customized contrast were MBB vs. SHE, *p* = .038, and MM vs. SHE, *p* = .096, respectively. None of the other three FF-MQ subscales were able to detect reliable differences among the three intervention groups.

It may be important to note that the MBB intervention did not include any *formal* mindfulness meditation exercises, while the MM intervention included several types of mindfulness meditation techniques (sitting, walking, and body scan). Nonetheless, MBB proved to be the intervention that increased the overall level of mindfulness in comparison with SHE.

##### Self-Compassion Scale

The MBB and MM treatments showed apparent benefit, with mean improvements from baseline levels of 7.25 (*p* = .002) for MBB and 4.38 (*p* = .046) for MM, but SHE showed virtually no change (.177, *p* = .939), in overall postintervention level. ANCOVA of total Self-Compassion scores yielded *p* = .084 and $R_{\beta}^2$ = .089, overall. The adjusted means and 95 % CIs of the three groups are presented in Table 2. The customized contrasts were MBB vs. SHE, *p* = 028, and MM vs. SHE, *p* = 160, respectively. Figure 4c presents these adjusted means graphically. Table 7 presents unadjusted means and standard deviations of this measure. This suggests that MBB was more effective than SHE in increasing self-reported measures of self-compassion (see Table 3 for estimated effect sizes involving the SCS).

##### WHO Well-Being Index

All treatment interventions showed benefit, with mean improvements from baseline levels of 4.96 (*p* = .001) for MBB, 3.77 (*p* = .001) for MM, and 2.70 (*p* = .001) for SHE, in overall postintervention levels. ANCOVA of total Well-Being Index (WBI) scores yielded *p* = .062 and $R_{\beta}^2$ = .095, overall. The adjusted mean scores and 95 % CIs of the three groups are presented in Table 2. The customized contrasts were MBB vs. SHE, *p* = 019, and MM vs. SHE, *p* = 267, respectively. Figure 4d presents these adjusted means graphically. Table 7 presents unadjusted means and standard deviations of this measure. This suggests that MBB was more effective than SHE in increasing self-reported measures of well-being (see Table 3 for estimated effect sizes involving WBI).

##### Positive and Negative Affect Schedule

Negative affect decreased in MBB (4.57), MM (3.79), and SHE (2.85) as indicated by adjusted mean scores. For positive affect, adjusted mean scores increased in MBB (3.46), MM (2.83), and SHE (1.0). However, the ANCOVA indicated that the groups did not statistically differ from one another for negative (*p* = .612) and positive affect (*p* = .278) (see Table 7 for unadjusted means and standard deviations of PANAS scores).

## Discussion

The present study provided preliminary evidence that cancer survivors can benefit from brief sleep-focused mind–body programs to improve their sleep. As hypothesized, MBB and MM performed better than SHE in decreasing self-reported sleep disturbance. Although both MBB and MM helped cancer survivors improve sleep, benefits seemed to emerge at different times. While MBB provided more benefit at program completion (at post), at 2-month follow-up, MM was found be equally as effective as MBB. This may be due to differential learning curves required for the two techniques. Data from the present study suggest that both MBB and MM can serve as effective interventions for sleep management in posttreatment cancer survivors.

In addition, MBB was found to have further benefits in comparison with the active control (SHE): (1) it decreased self-reported symptoms of depression, and (2) it increased overall levels of mindfulness, self-compassion, and well-being. These changes came about even though MBB was not specifically designed to explicitly address issues concerning these domains. The fact that MBB was effective in attenuating depressive symptoms was consistent with the similar pattern observed in our earlier study of MBB, although in the earlier study it did not reach statistical significance [33]. MM did not produce any reliable change in these secondary outcomes. These results provide preliminary support for the intriguing possibility that sleep-focused MBB can help cancer survivors not only with sleep disturbance but also with other comorbid symptoms such as depression.

In contrast, there were no reliable improvements in FACT-G, PSS, IES, and PANAS with either MBB or MM. As noted above, both MBB and MM produced improvements in FACT-G that are comparable to the magnitude of clinically meaningful changes reported in the literature [69], and this suggests that the mean improvements observed in the MBB and MM groups are in fact clinically meaningful by conventional standards. Similarly, the magnitudes of changes measured by IES and PSS in the MBB and MM groups are comparable to those reported in two recent studies of MBSR for cancer patients or survivors [52, 70]. Furthermore, it may be the case that these indicators (e.g., IES, PSS, PANAS) are more resistant to short-term changes.

Our interventions were designed to exclusively deal with cancer survivors’ problems concerning sleep. Given the possibility that improved sleep can influence other coexisting health conditions, we hypothesized that such sleep-focused interventions might also improve comorbid symptoms. Our objective was to explore the potential value of MBB and MM in improving self-reported sleep and comorbid symptoms in a cross-section of posttreatment cancer survivors, regardless of the type of sleep problems experienced or the origins of their sleep problems. Overall, the data are consistent with the hypothesis that MBB and MM can improve self-reported sleep quality in cancer survivors. However, we cannot yet claim that MBB or MM effectively treated specific sleep disorder conditions (such as insomnia) as assessed by clinical evaluation. Further research would need to explore potential benefits for specific sleep disorders such as primary, secondary, or comorbid insomnia, sleep apnea, restless leg syndrome, or sleep-disordered breathing.

The present study provided strong support for the idea that three weekly sessions of MBB or MM can yield meaningful sleep improvement. Our data are consistent with previous research using other interventions to improve sleep. Cognitive behavioral therapy also has been shown to be effective over a short-term period (1–4 sessions) [71, 72]. However, MBSR-type interventions may require longer durations (i.e., 6 sessions or longer) to exert their effects (for more on this issue see Carmody [73]), given that meditation practice might require a longer time span in order for it to generate sustained benefits.

The effectiveness of mindfulness practices in facilitating health and well-being, including sleep, has been reported in numerous studies utilizing several interventions [7, 17, 32, 74–78]. We postulated that increased mindfulness might have contributed to improvements in sleep and reductions in self-reported symptoms such as depression. Changes in mindfulness in the MBB group of this study are notable, especially given (1) the brief nature of the intervention (three sessions, once per week) and (2) the fact that, unlike other mindfulness-based interventions, MBB does not include formal meditation practice. However, MBB included everyday awareness practices, which are analogous to informal practice of mindfulness. This suggests the possibility that cancer survivors in the MBB group were able to engage in awareness practices without necessarily first going through formal mindfulness practices, in the form of sitting and walking meditation. The overall level of mindfulness increased in the MM group, but it was not statistically different from that in the control SHE group, despite the fact that MM improved sleep considerably.

Our exploratory study included participants in remission from a range of cancer types. Since this study assessed efficacy of MBB and MM as novel sleep management interventions, we did not exclude any specific sleep problems. Although it would be desirable to conduct an additional analysis using cancer type or specific sleep disorder as a covariate, the smaller sample size in the present study made it unfeasible to carry out this. Future studies with a bigger sample should investigate this issue. Nonetheless, given positive improvements from the two mind–body intervention groups, these inclusive selection criteria may actually contribute to the enhanced generalizability of our findings to the larger cancer survivor population. These results have important implications for selectively targeting sleep in cancer survivors, with potential additional benefits for comorbid symptoms such as depression.

In this study, MM did not produce beneficial changes in other indicators of psychosocial functioning. This may relate to the question of what “dose” of a mind–body intervention is required to produce observable benefits. MBSR has usually been implemented as an 8-week program since its inception; more recently, sleep-focused MBSR has been implemented as a 6-week program. In our study, MM was given in just three sessions over 3 weeks in accordance with the duration of the MBB program. This may have limited the efficacy of MM, and it would be reasonable to suspect that more sessions given over a longer time period might have made MM more effective in increasing MM’s impact on these other psychosocial indicators.

The present exploratory study produced some encouraging findings, but we are keenly aware of limitations that should be addressed in future clinical trials of MBB and MM. Limitations of our study include the following: the investigative method used to verify the mental health and remission status of study participants; no clinical (i.e., medical) evaluation of study participants with respect to the nature of sleep disturbances and other comorbid conditions (cancer survivors participating in the study, however, had been seeing their health care providers for any posttreatment problems); exclusive reliance on self-report measures; no explicit assessment of intervention fidelity; the lack of an explicit measure of home practice in the MM and MBB groups; and no measures of daily changes in sleep patterns based on sleep diary data.

Recently, Garland and her colleagues [79] proposed a randomized controlled noninferiority trial in which MBSR will be compared with Cognitive Behavior Therapy for Insomnia (CBT-I, a known efficacious treatment). This design can efficiently compare these two treatments directly and determine whether MBSR performs to the same standard as CBT-I for the treatment of insomnia with additional benefits of reducing cancer-related comorbid symptoms. Employing this type of study design may prove to be useful in further investigating MBB together with other proven treatments such as CBT-I.

The present exploratory study suggest that MBB and MM can serve as effective mind–body interventions for posttreatment care of cancer survivors with self-reported sleep disturbance. Furthermore, because MBB in particular is easy to learn and cumulative benefits are obtained within only a few sessions, MBB may be an ideal vehicle for managing multiple coexisting symptoms in cancer survivors. However, more research is clearly needed to determine if MBB may specifically serve as a front-loaded program for addressing sleep problems and comorbid psychological conditions like depression in posttreatment cancer survivors. Additionally, future studies might investigate the degree to which mind–body interventions may enable cancer survivors to reduce use of sleep medication. MBB in particular, and MBIs in general, can empower cancer survivors to manage complex symptoms, enhance quality of life, and regain more optimal functioning.
